# Assessment of material identification and quantification in the presence of metals using spectral photon counting CT

**DOI:** 10.1371/journal.pone.0308658

**Published:** 2024-09-13

**Authors:** Briya Tariq, Osama Sikander, Nadine Francis, Manar Alkhatib, Farhat Naseer, Naoufel Werghi, Esat Memisoglu, Nabil Maalej, Aamir Raja

**Affiliations:** 1 Department of Physics, Khalifa University, Abu Dhabi, United Arab Emirates; 2 Department of Biomedical Engineering & Sciences, National University of Sciences and Technology, Islamabad, Pakistan; 3 Department of Robotics and Intelligent Machine Engineering, National University of Sciences and Technology, Islamabad, Pakistan; 4 Department of Electrical & Computer Engineering, Khalifa University, Abu Dhabi, United Arab Emirates; 5 Imaging Institute, Cleveland Clinic Abu Dhabi, Abu Dhabi, United Arab Emirates; Politecnico di Torino, ITALY

## Abstract

Spectral Photon Counting Computed Tomography (SPCCT), a ground-breaking development in CT technology, has immense potential to address the persistent problem of metal artefacts in CT images. This study aims to evaluate the potential of Mars photon-counting CT technology in reducing metal artefacts. It focuses on identifying and quantifying clinically significant materials in the presence of metal objects. A multi-material phantom was used, containing inserts of varying concentrations of hydroxyapatite (a mineral present in teeth, bones, and calcified plaque), iodine (used as a contrast agent), CT water (to mimic soft tissue), and adipose (as a fat substitute). Three sets of scans were acquired: with aluminium, with stainless steel, and without a metal insert as a reference dataset. Data acquisition was performed using a Mars SPCCT scanner (Microlab 5×120); operated at 118 kVp and 80 μA. The images were subsequently reconstructed into five energy bins: 7-40, 40-50, 50-60, 60-79, and 79-118 keV. Evaluation metrics including signal-to-noise ratio (SNR), linearity of attenuation profiles, root mean square error (RMSE), and area under the curve (AUC) were employed to assess the energy and material-density images with and without metal inserts. Results show decreased metal artefacts and a better signal-to-noise ratio (up to 25%) with increased energy bins as compared to reference data. The attenuation profile also demonstrated high linearity (R^2^ >0.95) and lower RMSE across all material concentrations, even in the presence of aluminium and steel. Material identification accuracy for iodine and hydroxyapatite (with and without metal inserts) remained consistent, minimally impacting AUC values. For demonstration purposes, the biological sample was also scanned with the stainless steel volar implant and cortical bone screw, and the images were objectively assessed to indicate the potential effectiveness of SPCCT in replicating real-world clinical scenarios.

## Introduction

Metal artefacts in computed tomography (CT) obstruct visualization and assessment of anatomical structures, leading to inaccurate patient diagnoses and treatments. These artefacts occur in the presence of various metal objects; such as intracranial coils, clips, stents, hip or knee prosthetics, dental implants, and other surgical instruments. They may eventually hinder the evaluation of tissues near or within metal structures [1–3].

Metal related artefacts typically arise when metal objects attenuate X-rays, resulting in bright and dark streaking bands in the reconstructed images that cause inaccuracies in CT numbers. These discrepancies might extend beyond the immediate vicinity of metal-containing regions, impacting areas without metallic objects as well [4–9]. In general, streaks and cupping artefacts are common occurrences in CT imaging, typically caused by a combination of factors including beam hardening (which refers to the alteration of the X-ray spectrum as it passes through the object) and photon starvation (resulting from attenuation of X-rays by dense materials like metal). Additionally, other factors such as the partial volume effect (wherein the presence of multiple materials within a single voxel leads to inaccuracies in overall voxel value) and scattering (deviation of X-rays from their original path due to interaction with high atomic number materials and dense metal objects) are also considered as significant contributors to metal-related artefacts [2, 8].

While several studies focus on the development of dedicated image reconstruction and post-processing algorithms for reducing the severity of metal artefacts [10–15], some studies have also reported that their usage may introduce inaccuracies in CT numbers and result in new residual artefacts [4, 16–19]. Other studies have also investigated different methods to reduce metal artefacts at the data acquisition stage [7, 20–22]. Zhou et al. reported one such method by elevating the X-ray tube voltage and the current–time product to address the challenge of insufficient photons reaching the detector, albeit at the cost of increased radiation doses [23]. Alternatively, the incorporation of a tin filter enhances dose efficiency by boosting the proportion of high-energy photons in the spectrum [24]. Another approach acknowledged the effectiveness of virtual mono-energetic images from dual-energy CT at high mean energy (110–150 keV) [19, 25–27]. All these techniques demonstrate that existing methods for reducing metal artefacts are effective to some extent. However, there remains a clinical need for further enhancement. Therefore, this study investigates the potential of reducing metal artefacts at the data acquisition level using a small pixel photon-counting detection system incorporated Mars spectral photon-counting CT (SPCCT).

Mars SPCCT is an advanced medical imaging technique that combines the principles of conventional CT with spectral imaging using pixel-counting detectors (PCDs). PCDs employ direct conversion technology for X-rays using semiconductors, such as silicon (Si), Cadmium Telluride (CdTe), and Cadmium Zinc Telluride (CZT). Key features of the PCDs include their direct conversion technology using small pixel detectors, reduced charge sharing effect, provision of higher energy and spatial resolution, as well as eliminating the electronic noise through adjusting the energy thresholds above the noise floor of the chip [28]. Depending on the application-specific integrated circuits (ASIC) designs, PCDs can incorporate multiple energy thresholds, typically ranging from two to eight. This enhances the accuracy and precision of material identification and quantification using a distinctive K-edge discontinuity of high-Z contrast agents [29–36] and diminished beam-hardening artefacts in the presence of metal implants [7, 29, 37].

Several studies show the quantitative analysis of metal artefacts in energy images. However, to the best of our knowledge, the simultaneous evaluation of multi-energy images and material images in the presence of metal artefact to characterize near-metal visibility has not been previously quantified. Therefore, this paper aims to demonstrate how SPCCT technology can quantify metal artefacts across multi-energy bins, and material images in the presence of metal objects. This involves characterizing parameters such as the signal-to-noise ratio, spectral and linearity responses, area under the curve (AUC), and root mean square error (RMSE). For demonstration purposes, a bovine femur bone was also employed as a biological sample. Furthermore, a stainless steel volar implant was placed to assess the reduction in metal artefact and its impact on bone density as well as the visualization of the bone-metal interface.

## Materials and methods

### Phantom and biological specimen configuration

**Fig 1:**
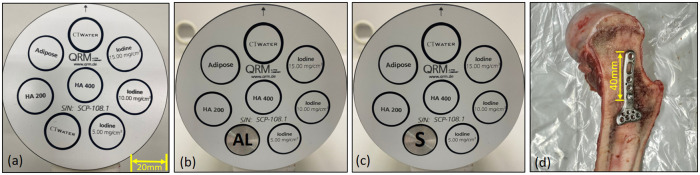
QRM phantom variations and bovine femur biological sample. The image of a 100-mm-diameter QRM phantom without any metal inserts for reference data (a), with an aluminium (AL) insert (b), and with a stainless steel insert (S)(c). The phantom has two calibration rods of HA (201.4 and 406.9 mg/cm^3^), and three rods of iodine (4.83, 9.66, and 14.56 mg/cm^3^) along with adipose and water. The biological sample is a bovine femur with a stainless steel volar fixation plate and cortical screw (d).

The study used a 100-mm-diameter QRM spectral CT phantom (QRM GmbH, Moehrendrof, Germany) with eight 20-mm-diameter holes to accommodate multiple solid inserts of tissue-equivalent materials and/or contrast agents. We used two concentrations of calcium hydroxyapatite (HA) (201.4 and 406.9 mg/cm^3^ to mimic the calcium and phosphate-rich areas in bone) and three concentrations of iodine (4.83, 9.66, and 14.56 mg/cm^3^ to simulate contrast agent) along with adipose insert (as a fat substitute) and CT water (as soft tissue). Three distinct datasets were obtained: one without metal inserts for reference (Fig 1a); also used for material decomposition calibration purposes, one with an aluminium (AL) insert (99% AL; 20-mm-diameter) (Fig 1b), and another one with a stainless steel insert (surgical stainless steel; 20-mm-diameter) (Fig 1c). For illustrative purposes, a bovine femur bone (from a supermarket) was used as the biological specimen. A 57-mm-long stainless-steel volar fixation plate and 20-mm-long stainless steel cortical screw (VLBPL-5–7, TriMed, Inc., Valencia, USA) were positioned in the trabecular part of the bone. The 3.1-mm-diameter cortical screw was inserted into the distal hole of the plate (thickness = 2.4 mm) to provide support and prevent any displacement of the plate within the bone (shown in Fig 1(d)).

### Mars scanner acquisition configuration

Imaging was conducted using a small-bore Mars SPCCT scanner (Mars Microlab 5×120). The scanner features a Mars camera with a 16.8 cm × 1.4 cm imaging array of Medipix3RX ASIC, bump-bonded onto 2 mm thick CZT at 110 *μ*m pitch. CZT demonstrated high detection efficiency within clinical diagnostic X-ray energies (>95% at 80 keV and >70% at 120 keV), making it suitable for spectral imaging. A bias voltage of ‐750V was applied across the CZT sensor layer. The scanner incorporates a microfocus poly-energetic X-ray source (SourceRay SB-120–350, SourceRay Inc., Bohemia, NY) with a tube voltage up to 120 kVp. The X-ray source is equipped with 1.8 mm AL equivalent intrinsic filtration and 0.125 mm external brass filtration. The scanner bore has a 120-mm-diameter AL tube with a 1.6-mm thickness. Both the source and the Mars camera are affixed to an internal gantry rotating around a stationary sample bed, maintaining a 285 mm source-to-detector distance and a 208 mm source-to-object distance. The scanner performs up to eight energy bin acquisitions simultaneously, achieving enhanced energy resolution through the utilization of Medipix3RX feature for interpixel communication in charge summing mode. This effectively reduces the impact of the charge-sharing effect in the four charge-summing counters and the arbitration counter (set at approximately 7 keV, above the noise floor of the Medipix3RX chip). Energy calibration was performed using threshold scans of the Bremsstrahlung spectrum for various anode voltages (kVp) [38, 39].

All scans were performed in charge summing mode with default thresholds of 40, 50, 60, and 79 keV. The tube current was set at 80 *μ*A to maintain a photon count rate of less than 11 counts/ms, based on previous studies, to avoid detector saturation and pulse pileup [40]. A total of 981 circular projections were captured for every 360° gantry rotation in a helical scan, with an exposure time of 160 ms per frame. In this study, no specialized metal artefact reduction software was employed as the vendor has not yet introduced dedicated software for metal artefact reduction. The biological specimen was also scanned using the same acquisition parameters described earlier. Before scan acquisition, a reconstruction mask was created using default vendor settings based on dark‐field images (20 frames per scan) for dark‐field correction and open-beam images (200 frames per scan) for flat-field normalization. During image reconstruction, this mask was applied to exclude unreliable pixels from the reconstruction process to minimize the ring artefacts. The spectral data were reconstructed in narrow energy bins using a proprietary iterative reconstruction algorithm (7–40, 40–50, 50–60, 60–79, and 79–118 keV) [31, 32, 41]. Subsequently, the reconstructed images were transferred to the built-in PACS for data visualization and further analysis. The resulting images had an isotropic voxel size of 0.1 mm and an image matrix of 1260 x 1260.

### Energy image analysis

The spectroscopic response for each concentration of HA and iodine was evaluated voxel by voxel using box-and-whisker plots, with regions of interest (ROIs) each consisting of 1500 voxels (150mm^2^). These ROIs were consistently positioned across all five energy bins. Ten random CT slices were analyzed, and the values were averaged for each data point in the box plot. For Hounsfield units (HU) calibration, the average linear attenuation values were converted into spectral HU with air (HU = -1000) and water (HU = 0) values using Eq 1.

$$\begin{array}{c} HU(E)=\frac{\mu_{mat}\left(E\right)-\mu_{water}\left(E\right)}{\mu_{water}\left(E\right)-\mu_{air}\left(E\right)}\times 1000 \end{array}$$ (1)

*μ*_mat(*E*)_, *μ*_water(*E*)_, and *μ*_air(*E*)_ are the linear attenuation coefficients of the material of interest, water, and air, respectively at energy *E*. For linearity assessment, the influence of X-ray signal intensity on material concentration was plotted and quantified across all five energy bins. The assessment was performed using a linear regression technique. To rule out systematic bias or random error in measurements and evaluate pair-wise proportional bias along with the limit of agreement, SNR assessment was performed using Bland-Altman plots and divided into three categories for each dataset: within the material rods, in the immediate vicinity of the metal inserts, and the outer proximity of the metal insert (phantom body). Ten random CT slices were analyzed on average for each category using four circular ROIs, each consisting of 560 voxels (56 mm^2^). SNR values for each ROI were derived across all five energy bins by calculating the ratio of the mean linear attenuation values to their standard deviation (as a measure of noise and metal artefacts). SNR was also statistically evaluated across all energy bins for datasets with AL and steel inserts, and compared with the reference data (without any metal inserts). For statistical assessment, the student t-test was applied on the average of SNR values across four ROIs and the level of significant difference between datasets at p<0.05 was determined.

### Material image analysis

A typical approach for a material decomposition (MD) algorithm converts spectral attenuation into material-specific identification and quantification using basis material maps. This process is based on material-specific energy-dependent attenuation, the atomic composition of the material, and the material’s density. Furthermore, material densities are directly related to the linear attenuation of the relevant material through mass attenuation (*μ*/*ρ*_*mE*_). In this study, we applied vendor-provided material decomposition software (MARS-FASTMD v1.4)which is based on a constrained linear least squares technique [42, 43]. Such MD requires prior knowledge which is usually measured through material phantoms to estimate the mass attenuation basis of target materials. This information then serves as input for the material density estimation. In general, the energy-specific linear attenuation of a composite material can be written as a linear combination of the material constituents given in Eq 2.

$$\begin{array}{c} \mu \left(E\right)=\sum_{m}x_{m}{\left(\frac{\mu }{\rho }\right)}_{\text{m}}^{\text{E}} \end{array}$$ (2)

Whereas *μ*(*E*) is the composite linear attenuation coefficient function of multi-energy data, *x*_*m*_ is the density or concentration of *m* material, and ${\left(\frac{\mu }{\rho }\right)}_{\text{m}}^{\text{E}}$ is the mass attenuation for material *m* at energy *E*. Additional information about the Mars MD can be found in Bateman et al [43]. In our study, the reference data (without metal inserts (Fig 1a)) was used for MD calibration purposes to calibrate the other two phantoms (Fig 1(b) and 1(c)). The ex-vivo bovine sample was decomposed into material components using a separate reference phantom (figure not shown here) with five concentrations of HA (49.2, 102.4, 201.5, 406.9, 809.8 mg/cm^3^) along with adipose and water.

#### Evaluation metrics

The quantitative evaluation of material identification and quantification for HA and iodine in all three datasets was carried out using an automated strategy developed in Python (Python 3.10.12). The quantitative metric provides a true positive rate in terms of sensitivity and a true negative rate in terms of specificity. For comparative assessment, the dataset without a metal insert served as a reference for the other two datasets that contained AL and steel inserts. For the sensitivity of the target material in the density image domain (such as HA density image), the total number of voxels within the selected ROI, comprising 1500 voxels (150 mm^2^) was compared to the voxel count in the equivalent ROI of the energy image (the ground truth). Voxels correctly recognized as the target material were classified as true positives (TP), while those that were not recognized were classified as false negatives (FN). To evaluate specificity, the ROI of the entire phantom area in the same density image consisting of 7850 mm^2^ was compared with the ROI of the target material. The false positive value (FP) represents the total number of voxels within the density image that appeared as material other than the target. The true negative value (TN) refers to the total number of voxels correctly not identified as anything other than the target material. AUC was calculated using the trapezoidal rule using Eq 3. To calculate the AUC, the true positive rate (TPR) and the false positive rate (FPR) were evaluated over a range of quantified concentration thresholds between their upper and lower quartiles. For a more in-depth quantitative assessment accuracy, positive predicted value (PPV), and negative predicted value (NPV) were also evaluated.

$$\begin{array}{c} \text{AUC}=\sum_{i=1}^{n-1}\frac{\left({\text{FPR}}_{i+1}-{\text{FPR}}_{i}\right)\left({\text{TPR}}_{i+1}+{\text{TPR}}_{i}\right)}{2} \end{array}$$ (3)

TPR represents sensitivity and FPR indicates the value of 1-specificity. *i* represents the thresholds over the range of qualified concentration.

## Results

### Spectral and linearity response

**Fig 2:**
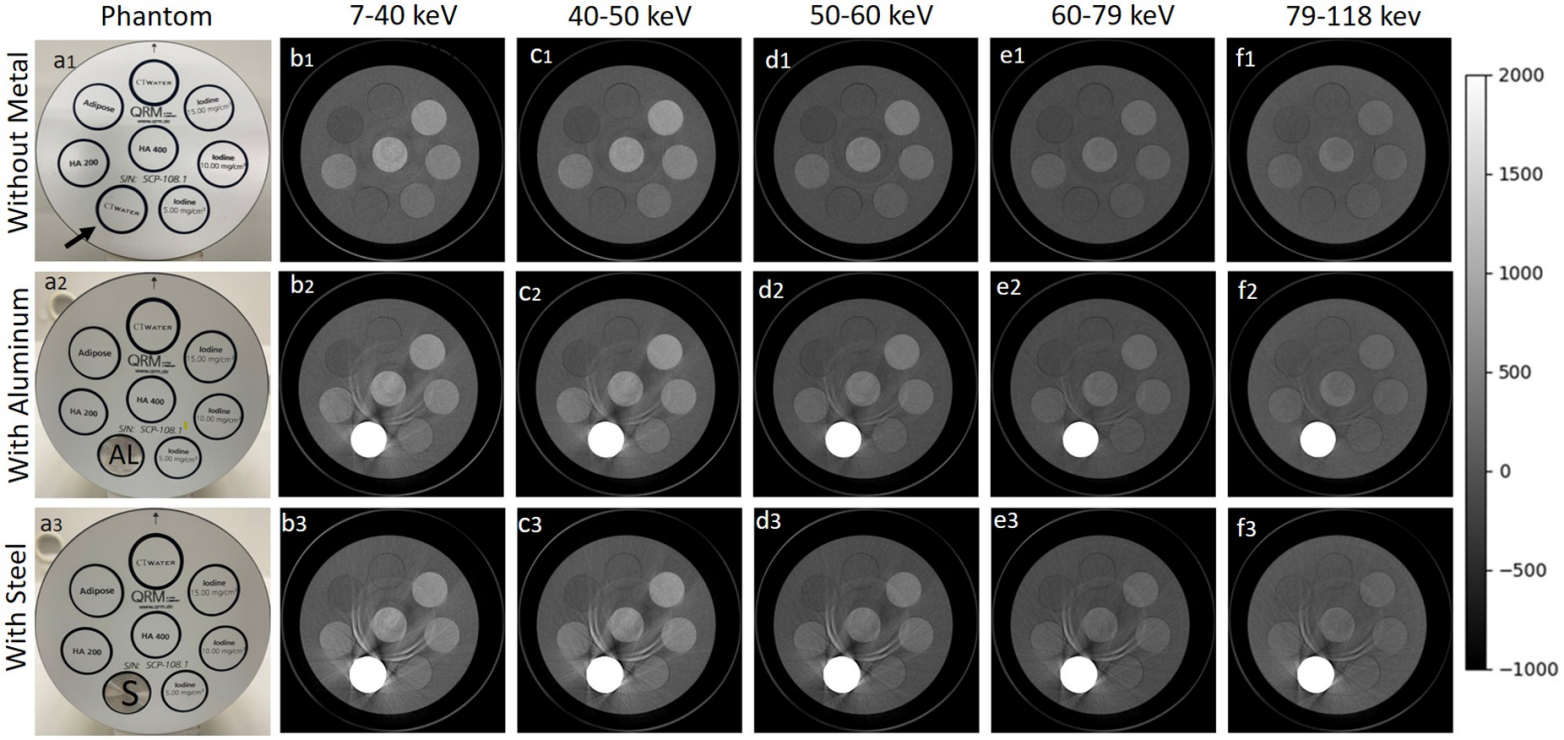
Spectral images for three datasets. Illustration of the phantom image (a) and its CT images acquired at 118 kVp and 80 μA across five energy bins (b,c,d,e,f). The phantom without any metal insert (a1), with aluminum (AL) (a2), and with stainless steel (a3), has two calibration rods of HA (201.4 and 406.9 mg/cm^3^), three rods of iodine (4.83, 9.66, and 14.56 mg/cm^3^) along with adipose and water rods each 20-mm in diameter. Energy images of metal inserts reveal the metal artefacts reduction in higher energy bins. The grayscale bar represents the Hounsfield units (HU) range from -1000 to 2000.

**Fig 3:**
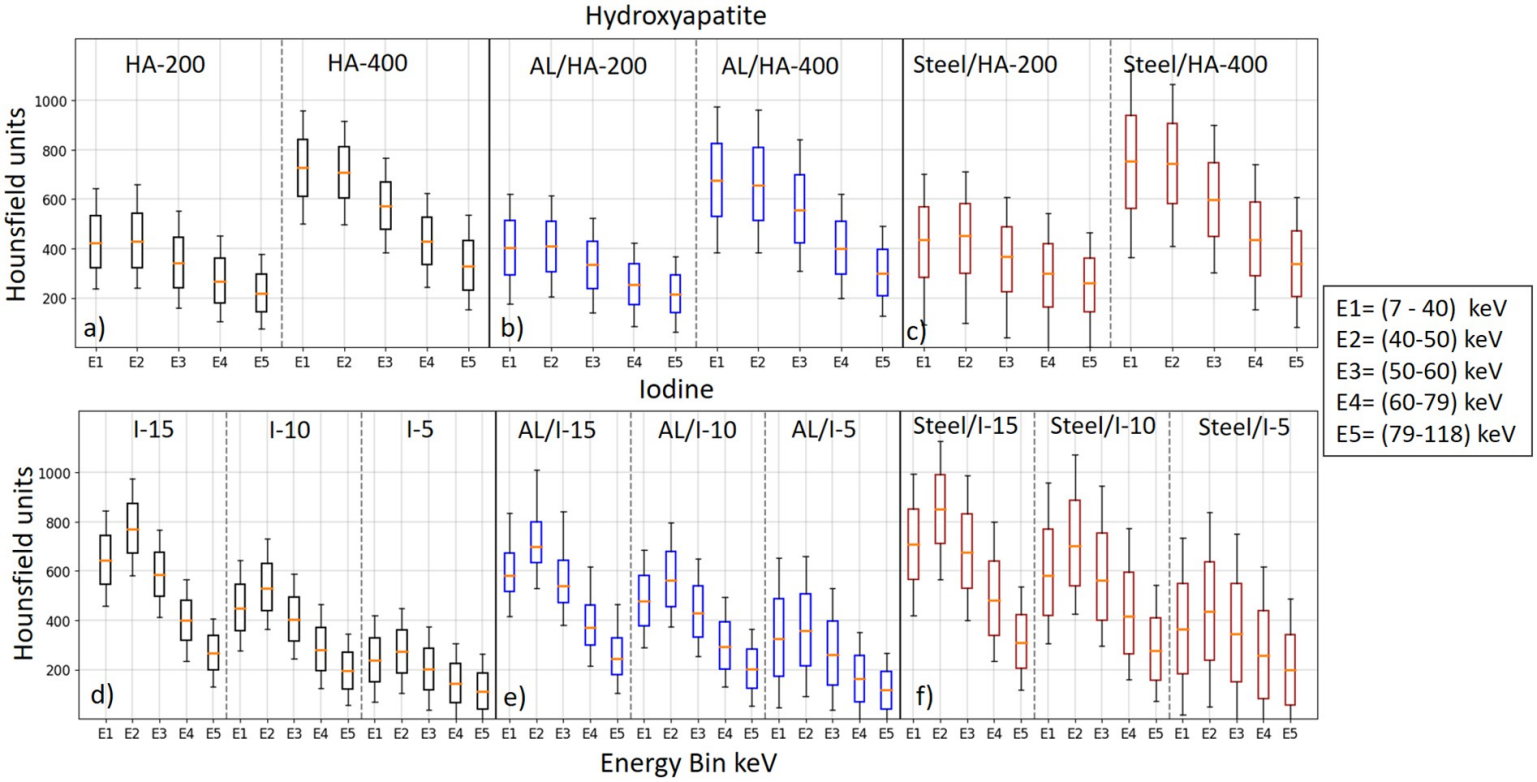
Voxel-wise spectral response. The top row shows the Voxel-wise spectral response of HA (201.4 and 406.9 mg/cm^3^) without metal insert (a), with aluminium (AL) (b), and with stainless steel (c). The bottom row shows the spectral response of iodine (4.83, 9.66, and 14.56 mg/cm^3^) without metal insert (d), with aluminium (AL) (e), and with stainless steel (f). The horizontal line inside each box represents the median value (50% percentile of the data). The top and bottom boundaries of the box indicate the lower and upper quartile values of HU representing the 25% and 75% percentiles respectively.

**Fig 4:**
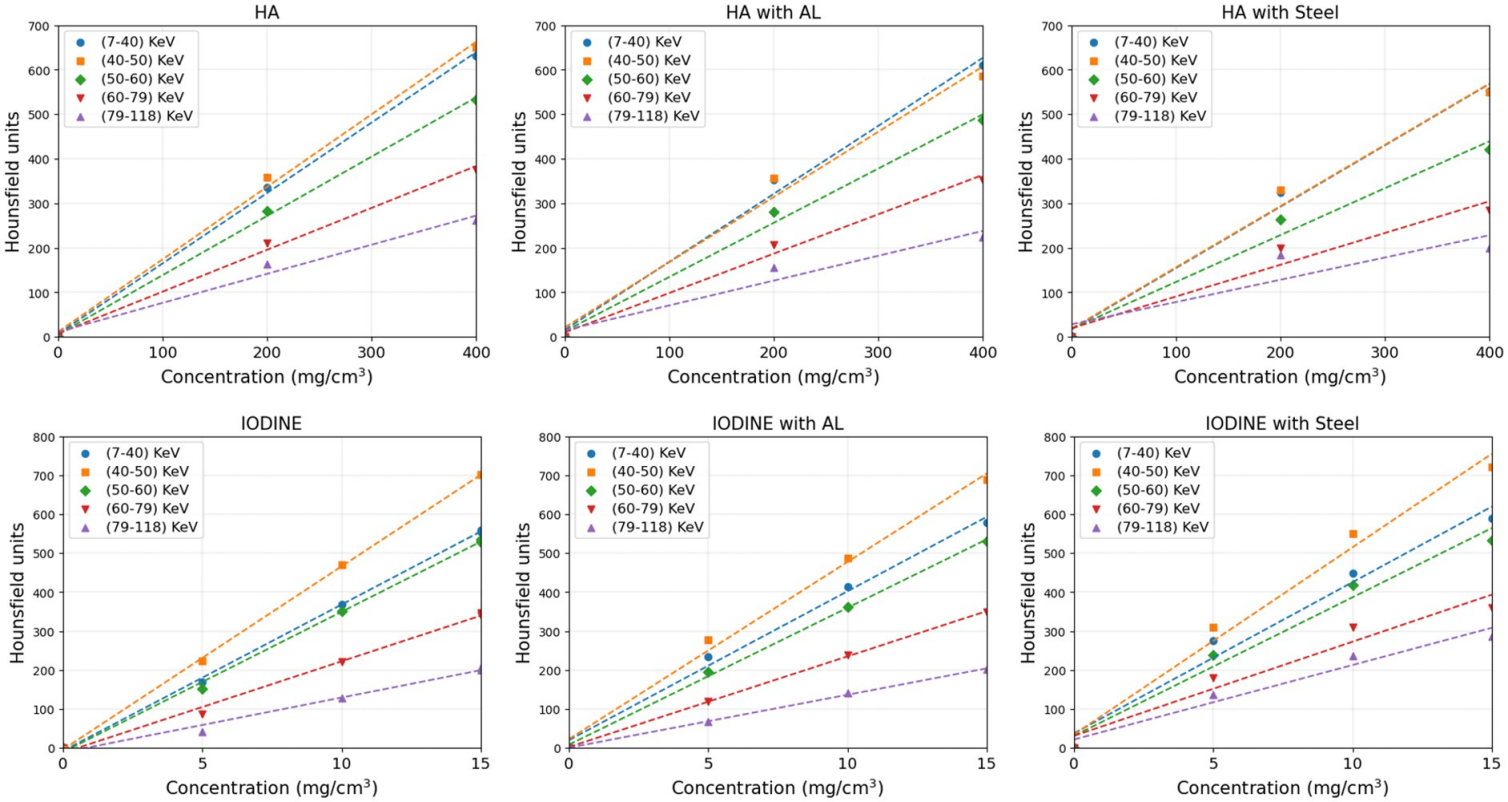
Linearity response of x-ray attenuation. Linear regression response of x-ray attenuation (HU) in each energy bin as a function of known concentrations of hydroxyapatite (HA) (a–c) and iodine (d–f) in the absence and presence of aluminium (AL) and steel. The standard error ranges between 1 to 3 HU.

Overall, the grayscale multi-energy images in Fig 2 show streak artefacts in the presence of AL and stainless steel, and ring artefacts occur primarily as a result of leftover variation in gain among detector pixels after pixel masking. However, as expected, the artefact volume for both metals was significantly less in the higher energy bins compared to the lower energy bins. Particularly in the presence of AL artefacts almost disappeared at the highest energy bin (shown in Fig 2(f2)). Furthermore, steel-induced artefacts are more severe across all energy bins compared to AL-induced artefacts. Fig 3 shows the box-and-whisker plots to demonstrate the voxel-wise spectral response of three datasets comprising HA (201.4 and 406.9 mg/cm^3^) and iodine (4.83, 9.66, and 14.56 mg/cm^3^), both in the absence and presence of metal inserts. The voxel-wise information of 1500 voxels depicts the HU values, showcasing the median, lower quartile, and upper quartile for each dataset across all energy bins. Fig 4(a)–4(f) shows the linearity response of the system. It is characterized by the linear regression model for known concentrations of HA and iodine across all five energy bins, in the absence and presence of metal objects. The linear regression correlation (R^2^) and the accuracy of the linear model were quantitatively assessed by the root-mean-squared error (RMSE) to evaluate the predictive performance of the model, as summarized in Table 1. Achieving a strong correlation and minimal error across all energy bins is essential for ensuring accurate material identification and quantification.

**Table 1:** The linear regression (R^2^) and Root Mean Square Error (RMSE) values for Hydroxyapatite (HA) and iodine across each dataset.

**Energy bin (KeV)**	**R^2^ (HA)**			**RMSE (HA)**		
	**Without Metal**	**With AL**	**With Steel**	**Without Metal**	**With AL**	**With Steel**
**(7–40)**	0.99	0.99	0.98	2.35	2.25	2.10
(40–50)	0.99	0.98	0.98	2.44	2.25	2.11
(50–60)	0.99	0.99	0.97	1.98	1.85	1.63
(60–79)	0.99	0.99	0.94	1.42	1.35	1.15
(79–118)	0.97	0.97	0.80	1.07	0.89	0.89
**Energy bin (KeV)**	**R^2^ (Iodine)**			**RMSE (Iodine)**		
	**Without Metal**	**With AL**	**With Steel**	**Without Metal**	**With AL**	**With Steel**
**(7–40)**	0.99	0.99	0.97	2.27	2.46	2.60
**(40–50)**	0.99	0.99	0.98	2.87	2.92	3.15
**(50–60)**	0.99	0.99	0.97	2.16	2.21	2.36
**(60–79)**	0.99	0.99	0.97	1.38	1.44	1.67
**(79–118)**	0.98	0.99	0.95	0.81	0.84	1.30

### Signal-to-noise ratio (SNR)

**Fig 5:**
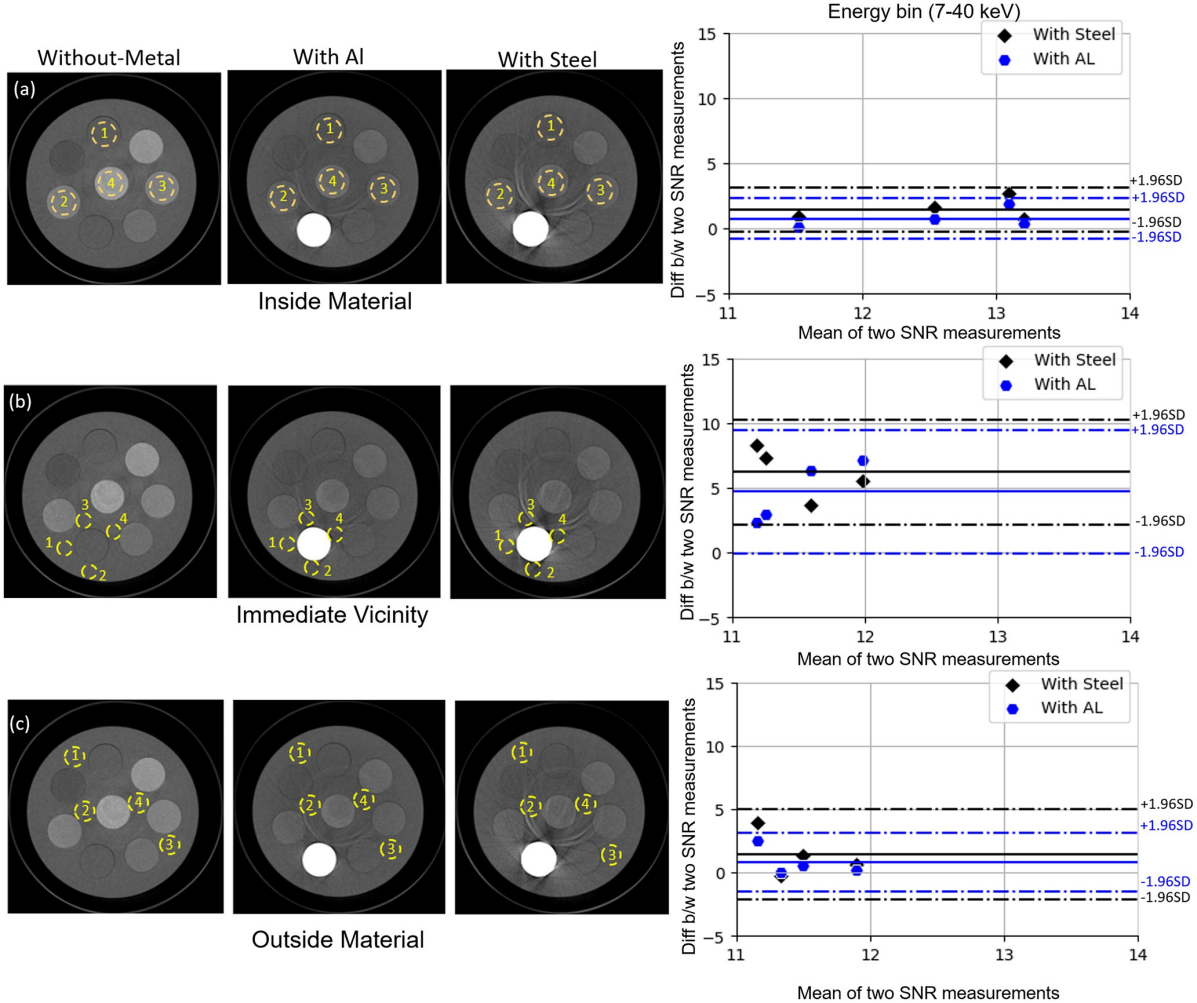
Pairwise assessment of signal-to-noise-ratio (SNR). Bland-Altman plots in the presence of aluminium (AL) and steel inserts as compared to reference data (without any metal insert) across four ROI (yellow circles) in each category: (a) inside the materials, (b) the immediate vicinity of metal, (c) and the outer proximity. Bland-Altman plots show the difference between (b/w) SNR (SNR without metal—SNR with metal) as a function of the mean SNR for each category (a,b,c). The solid blue and black lines represent the SNR difference for aluminium (AL) and steel, respectively. The corresponding dashed lines represent their upper and lower limits (confidence limits ± 1.96 × standard deviation.).

As energy levels increase, SNR exhibits a significant improvement of 35% and 18% in the immediate vicinity of AL and steel respectively, as compared to reference data. In the outer proximity, there is a rise in SNR by 18% and 30% for AL and steel, respectively. Bland-Altman plots in Fig 5 illustrate measurements for the first energy bin (7–40 keV) in all three categories: inside material, immediate vicinity, and outer proximity (shown in Fig 5(a)–5(c)). The y-axis shows the difference in SNR (SNR without metal—SNR with metal) while the x-axis shows the mean value of SNR with and without the metal. While all SNR measurements fall within the agreement limit range (± 1.96 × Standard Deviation), a greater difference is noticeable when comparing SNR with and without the metal insert, particularly in the immediate vicinity and outer proximity. The difference in SNR is slightly overestimated in the presence of steel compared to AL, attributing to the severity of artefacts in the presence of steel. The results also indicate that the average SNRs in the immediate proximity to both AL and steel inserts are highly significant (p<0.036 and p<0.001, respectively) than the reference data. Furthermore, in the outer proximity of AL and steel, the average SNR also demonstrates statistical significance (p<0.01 and p<0.004, respectively) than the reference data. Additional images related to the SNR analysis and Bland-Altman plots for all of the energy bins can be seen in S1–S4 Figs.

### Material identification and quantification

**Fig 6:**
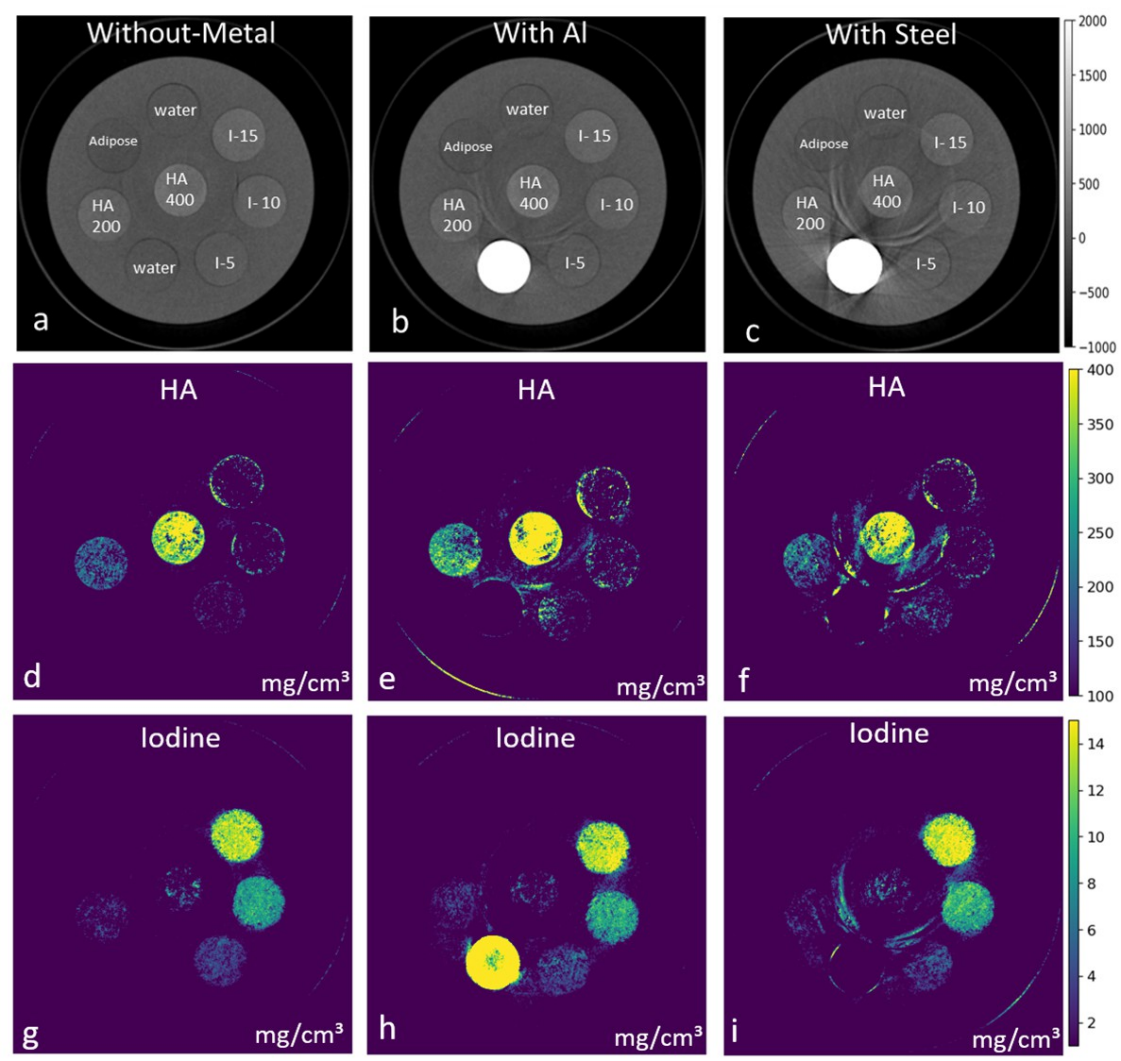
Material density images in the absence and presence of metal inserts. Energy images (top row; grayscale shows HU) and density images (second and third row; scale bar shows mg/cm^3^). Material decomposition eliminates the effect of aluminium (AL) and steel from the HA density profile (e, f). However, aluminium (AL) is misidentified as iodine (g) compared to steel (h).

**Fig 7:**
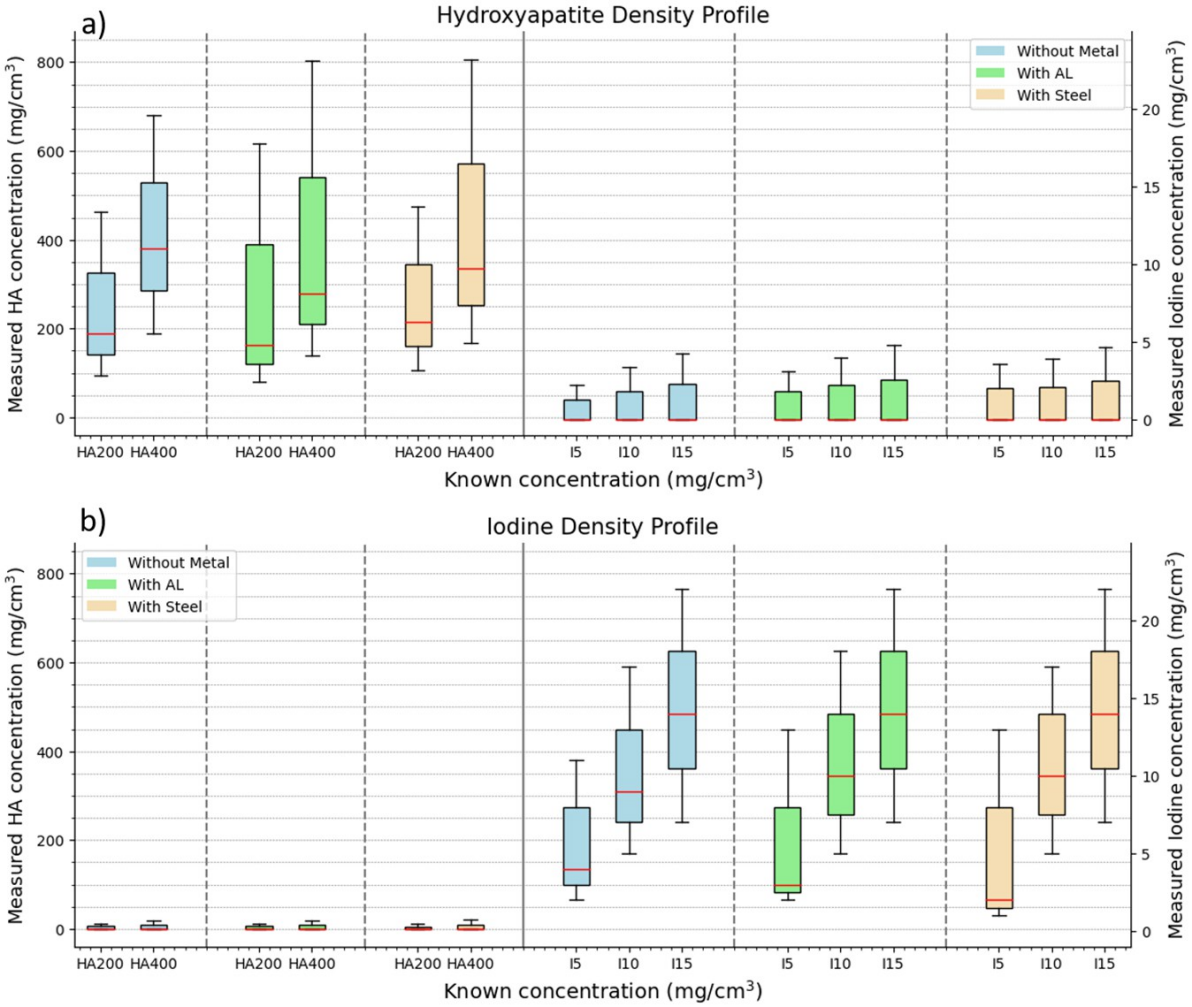
Voxel-wise comparison of measured concentrations as compared to known concentration. Voxel-wise comparison showing measured concentrations with known concentrations of hydroxyapatite (HA) (a) and Iodine (b). Dotted vertical grid lines separate datasets between those without metal inserts (blue), with AL (green), and with steel (yellow). In the HA density profile (a), the misidentification of iodine concentration and in the iodine density profile (b), the misidentification of HA is separated by solid vertical lines. Whiskers and boxes are described in the same manner as in Fig 3.

Phantom material density images of HA and iodine in the presence and absence of AL and steel are shown in Fig 6. The voxel-wise distribution of the measured concentration of HA and iodine as a function of known concentration, both in the absence and presence of metal inserts, shown in Fig 7(a) and 7(b) using box-and-whisker plots. The quantitative assessment shows that both HA and iodine concentrations are reasonably measured in their respective density profiles. However, some cross-talk between HA and iodine can also be observed. Performance parameters which include sensitivity, specificity, AUC, accuracy, negative predictive value (NPV), and positive predictive value (PPV) for material density images of HA and iodine, with and without metals, are summarized in Table 2. Overall, the metric parameter of sensitivity, specificity, and accuracy results of material identification for HA and iodine in the presence of metal inserts aligns well with the reference values (without metals) and demonstrate consistently high AUC levels (>74%) and accuracy levels (>85%). The quantification of material concentration for each component was quantified with low error (RMSE >0.29 mg/cm^3^) in both scenarios: with and without metal inserts.

**Table 2:** Characterization of material identification metrics (Sensitivity, specificity, Area under the curve (AUC 3), accuracy, Negative predictive value (NPV), and Positive predictive value (PPV)) and quantification analysis (root mean square error (RMSE)) for Hydroxyapatite (HA) and iodine, both with and without the presence of aluminium (AL) and steel.

Material (mg/cm^3^)	Material Identification						Material Quantification
	Sensitivity ($\frac{TP}{TP+FN}$%)	Specificity ($\frac{TN}{TN+FP}$%)	Accuracy ($\frac{TP+TN}{TP+TN+FP+FN}$%)	NPV ($\frac{TP}{TP+FP}$%)	PPV ($\frac{TN}{TN+FN}$%)	AUC %	RMSE* (mg/cm^3^)
**HA**	83	93	91	95	73	83	0.26
**HA with AL**	79	77	90	95	70	93	0.29
**HA with Steel**	78	93	90	93	75	75	0.21
**Iodine**	80	90	86	86	86	80	0.11
**Iodine with AL**	77	90	86	86	86	80	0.17
**Iodine with Steel**	79	90	85	86	84	74	0.11

TP, true positive; TN, true negative; FP, false positive; FN, false negative.

* RMSE values are presented as a percentage of the known concentrations.

**Fig 8:**
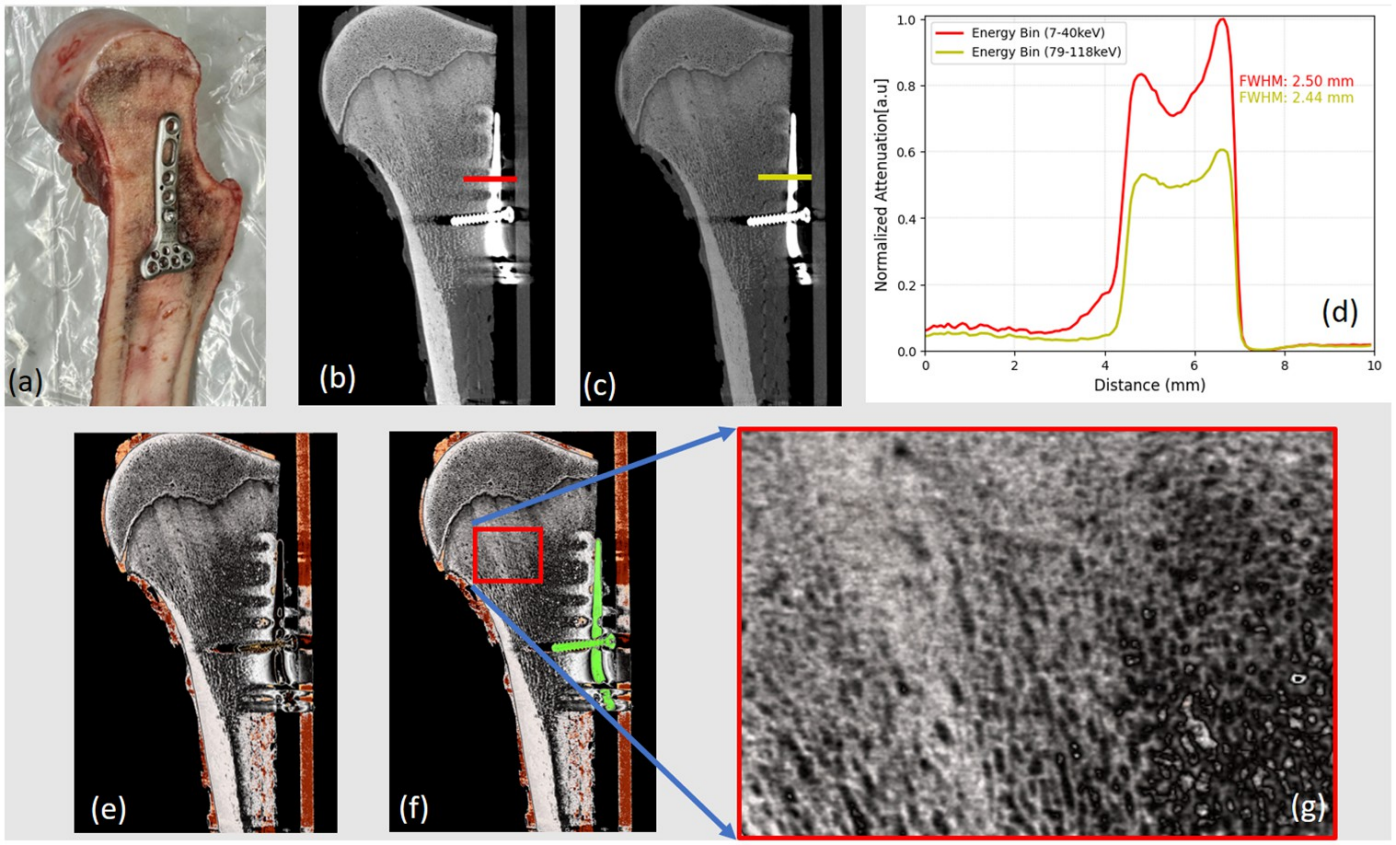
Illustration of biological sample. (a) Image of a biological sample (bovine femur bone) with a precisely positioned volar plate (wrist fixation plate) implant, along with a cortical bone screw made from stainless steel; (b) Sagittal view of a bone with an implant for the lowest energy bin (7–40 keV), (c) and higher energy bin (79–118 keV) with significantly reduced artefacts. (d) Normalized line profile for the lowest and highest energy bin showing a noticeable reduction in the artefacts. (e) The material density image demonstrates the differentiation of tissues: bone (white), fat (yellow), and soft tissue (orange-red), (f) accompanied by a combined image of material and energy, highlighting the implant in green. (g) Additional zoom image focuses on the bone density showing the capability of the SPCCT in detailing the structure of the bone.

Fig 8 shows a section of the biological sample (shown in Fig 8(a)) in the sagittal view (shown in Fig 8(b) and 8(c)) in the lowest and highest energy bin (7–40 keV and 79–118 keV, respectively). The images were generated using Mars Vision V2.5.6 software provided by MARS Bioimaging Ltd. As expected, in contrast to the lower energy bin, a reduction in the volume of streak artefacts within the proximity of the steel implant is evident in the higher energy bin. Furthermore, metal artefacts are eliminated at the higher energy bin, albeit with the trade-off of a diminished contrast difference between metal and bone. Nevertheless, the diminished contrast still achieves excellent differentiation between the metal and the non-metal regions. For the investigation of edge sharpness and the impact of metal artefacts on detecting the bone-metal interface and implant thickness (of 2.4 mm), line profiles were plotted for two energy bins (lowest and highest) as shown in Fig 8(d).

**Fig 9:**
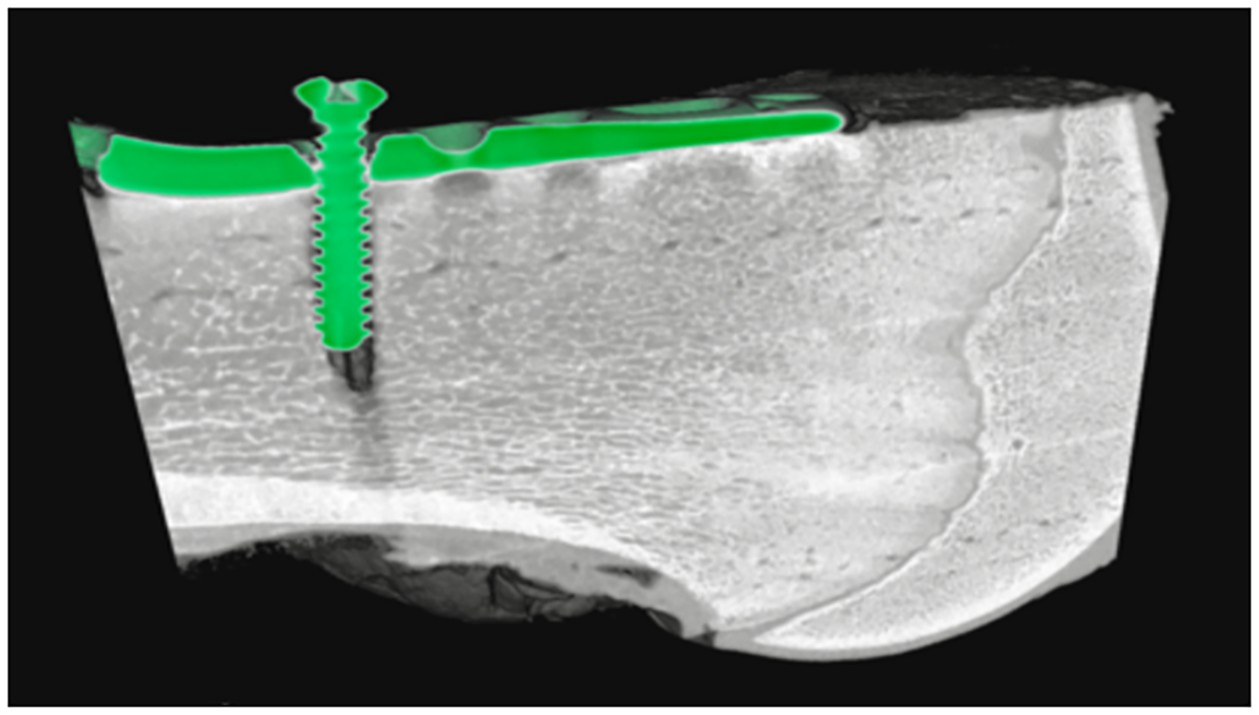
Three-dimensional rendering. Three-dimensional rendering of the bone sample using Mars visualization software. Green color showing the metal implant as volar fixation plate, and long stainless steel cortical screw.

The full width at half-maximum (FWHM) for these line profiles was calculated by measuring the width of the line profiles at half of their average maximum value [44]. Since the dataset exhibits two peaks, the average maximum value was determined by averaging the intensities of two peaks (FWHM = |*x*_2_ − *x*_1_|, where *x*_1_ and *x*_2_ are the two *x* values at which *f*(*x*) are equal to half of the maximum value). FWHM was measured at 2.50 mm for the 7–40 keV range and 2.44 mm for the 79–118 keV. When comparing these FWHM values with the actual thickness of the implant (2.4 mm), it became evident that metal artefacts in the lowest energy bin led to an overestimated metal thickness, potentially resulting in an apparent broadening of metal structures and impacting the accuracy of attenuation measurements at the bone-metal interface. Fused images of HA/bone (white), soft tissue (reddish-orange), and lipid (yellow) density images are shown in Fig 8(e). Fig 8(f) combines both material density images and energy images (79–118 keV) in which a green color map is assigned to the presence of a stainless steel implant. Fig 9 illustrates the 3D rendering of the bone sample. These images provide a clear visualisation of the interface between metal and non‐metal regions as well as the porosity of the bone sample.

## Discussion

In this work, a commercially available MARS SPCCT system incorporated with the Medipix3RX detector [28] was used for the simultaneous evaluation of multi-energy and material density images in the presence of metals to characterize near-metal visibility. Multi-energy images and material-density images both with and without metal inserts were assessed by spectral and linearity response, SNR, AUC, and RMSE.

Fig 2 shows that the severity of artefacts for both metals was significantly less in the higher energy bins compared to the lower energy bins. Furthermore, artefacts induced by the AL insert were less severe when contrasted with those caused by stainless steel. This could be attributed to the lower atomic number and density of AL (Z = 13; *ρ* = 2.7*g*/*cm*^3^, in comparison to stainless steel (Z = 26; *ρ* = 7.8*g*/*cm*^3^). Consequently, images with AL artefacts are less prone to issues, such as beam hardening, scattering, and photon starvation supporting the fact that the lower the atomic number of the metal, the lower the energy required to produce reduced streak artefacts. Furthermore, the box plots in Fig 3 indicate a wider range of voxel value variation across the energy bins in the presence of AL and steel. Steel exhibits broader voxel variation compared to AL and reference data. Additionally, As compared to the reference data, the attenuation values were overestimated in the presence of steel but were comparable in the presence of AL. A slight increase in the attenuation profile of iodine was observed in the energy bin 40–50 KeV, likely because of iodine’s K-edge. Other than the K-edge discontinuity of iodine, the attenuation values for both iodine and HA decrease for higher energy bins because of the diminishing effect of the photoelectric effect and the strong influence of the Compton scattering. This trend was consistent across all datasets.

The linearity response in Fig 4 of the system ensures the validity of the relationship between the CT number and electron density for a range of materials. The results in Table 1 show a reduction in RMSE values after the second energy bin (40–50 keV) for both HA and iodine across all three datasets. Notably, it was observed that the first energy bin is susceptible to underestimated RMSE values throughout the datasets. This underestimation may stem from various factors, encompassing beam hardening. However, an additional contributory factor may be linked to the behavior of the CdZnTe sensors. A significant proportion of pulses registered by the Medipix3RX ASIC at an energy pulse height below 30 keV originate primarily from the fluorescence photons of Cd (Z = 48; K-absorption edge = 27 keV) and Te (Z = 52; K-absorption edge = 32 keV). Despite the use of an additional 0.125 mm brass filter designed to exclude low-energy photons (approximately below 26 keV), various low-energy photons may still falsely contribute to the signal. SNR results in Fig 5 and S1–S4 Figs, demonstrate fewer streak artefacts and a significant increase in SNR (25%) in the higher energy bin (79–118keV) both in the presence of AL and steel. Other researchers have previously reported similar observations, indicating an overestimated SNR in the presence of metal implants due to streaking artefacts [45–47]. Furthermore, the average SNR in the immediate proximity of metal inserts is highly significant in the presence of steel (p = 0.003) as compared to AL (p = 0.017). However, in the outer proximity, improvement in the SNR in the presence of AL is more pronounced due to the inherent characteristic of AL as a less dense material, causing fewer artefact in comparison to steel.

The material density images of the phantom shown in Fig 6, eliminate the steel insert from the HA and iodine density image (shown in Fig 6(e) and 6(f)). However, AL was misidentified as iodine (shown in Fig 6(h)). This observation could be explained by the similarity in the cupping artefact (defined as the mean voxel values at sample edges divided by the central voxel value of the sample) between iodine (0.96%) and AL (0.94%), in comparison to HA (0.5%). Fig 7 shows a broader range of voxel-wise variation for HA and iodine concentrations in the presence of metals, as compared to the reference data. Nevertheless, the quantification results in the presence of metal inserts align well with the reference values (without metals) and demonstrate a consistently high AUC (>74%) and accuracy (>85%) with an RMSE <0.29%. However, a 5% and 6% decrease in the sensitivity and the AUC was recorded. In addition, a 4—8% increase in RMSE in the presence of metal was observed. The material discrimination abilities of spectral CT added with metal artefact reduction algorithms can further improve material visualization and quantification [48, 49]. These results also suggest that the presence of steel inserts introduces greater uncertainty in the accurate identification of materials compared to AL inserts, similarly reported by [45, 50].

Likewise, the examination of the ex-vivo bovine sample in Figs 8, and 9 shows the structural composition of the bone tissues in the presence of the stainless steel insert. The presence of artefacts in the lower energy bin widened the width of the steel implant compared to the higher energy bin. Furthermore, bone assessment involves understanding not only bone mineral density but also morphological factors like trabecular thickness, trabecular spacing, and cortical thickness (shown in Fig 8(g)). Subjective evaluation of biological images shows the potential of the Mars SPCCT technology in simultaneous measurement of both density and morphology even in the presence of metal implants (Fig 9).

There are a number of observations that can be drawn from this study. Firstly, the reduction of metal artefacts can be achieved at the acquisition level using Mars SPCCT technology without necessitating the introduction of numerical corrections. Secondly, the reduction of artefacts can be accomplished without resorting to extensive post-processing of the acquired datasets. Thirdly, the methodology is universally applicable for mitigating beam hardening artefacts in spectral scans containing dense high-Z materials-extending beyond scenarios involving the presence of metals. Lastly, the material identification and quantification capabilities of Mars technology exhibit potential enhancements in material visualization and quantification, particularly in samples featuring metal implants.

The results of this study have potential relevance to human imaging as we employed identical kVp and energy ranges currently being used in human point-of-care SPCCT for extremity scanners [23, 51]. However, it is important to acknowledge limitations in our study; namely the assessment of a limited number of metal inserts and the use of a single bovine specimen. Despite these constraints, our study successfully demonstrated the evaluation of energy and material images, enabling the assessment of metal artefact’s impact. In the future, this methodology holds the potential for comparing and optimising scanning protocols, refining image reconstruction methods, and enhancing techniques for material differentiation in spectral CT with or without metal inserts. Moreover, assessment of metal artefacts could be particularly valuable when integrating deep learning models with SPCCT data, as understanding the extent and nature of artefacts aids in designing improved ground truth datasets for deep learning models.

## Conclusion

This study demonstrates that SPCCT technology is an emerging tool for multi-energy imaging, particularly in metal artefact reduction at the acquisition level. However, this functionality extends beyond metal scenarios, effectively addressing beam-hardening artefacts with dense high-Z materials and contrast agents. In our study, the Mars SPCCT scanner displays stable linearity with improved accuracy in material characterization (as indicated by reduced RMSE values). Improved SNR, particularly in proximities, enhances image quality in the higher energy bins amid aluminium and steel presence.

## Supporting information

S1 Fig Signal-to-noise ratio analysis. Signal-to-noise ratio (SNR) values for the case without any metal insert (a), with steel (b), and with aluminum (c) divided into three groups; inside the material(a1,b1,c1), immediate vicinity (a2, b2, c2), and outside material (a3, b3, c3). SNR values in the immediate vicinity for steel and aluminum were observed statistically significant (p<0.05).
(TIF)

S2 Fig Pairwise assessment of SNR—Immediate vicinity. Bland-Altman plots showing the difference between SNR values as a function of the mean SNR values for the datasets of aluminum and steel inserts as compared to reference data (without any metal insert); for the case of the immediate vicinity of the metal object across five energy bins. The red and blue line represents the bias value, and the dashed lines represent the upper and lower limits of the mean values for steel and aluminum, respectively (confidence limits ± 1.96 × standard deviation).
(TIF)

S3 Fig Pairwise assessment of SNR—Inside material. Bland-Altman plots showing the difference between SNR values as a function of the mean SNR values for the datasets of aluminum and steel inserts as compared to reference data (without any metal insert); for the case of inside the material across five energy bins. The red and blue line represents the bias value, and the dashed lines represent the upper and lower limits of the mean values for steel and aluminum, respectively (confidence limits ± 1.96 × standard deviation).
(TIF)

S4 Fig Pairwise assessment of SNR—Outside material. Bland-Altman plots showing the difference between SNR values as a function of the mean SNR values for the datasets of aluminum and steel inserts as compared to reference data (without any metal insert); for the case of outside the material across five energy bins. The red and blue line represents the bias value, and the dashed lines represent the upper and lower limits of the mean values for steel and aluminum, respectively (confidence limits ± 1.96 × standard deviation).
(TIF)

S1 File Reference dataset. (ZIP)

S2 File Dataset with steel insert. (ZIP)

S3 File Dataset with aluminium insert. (ZIP)

S4 File Evaluation results. (ZIP)
