# Supplementary figures and images for: Assessment of material identification and quantification in the presence of metals using spectral photon counting CT
Source: PLoS One. 2024 Sep 13;19(9):e0308658. doi: 10.1371/journal.pone.0308658 (PMC11398698; doi:10.1371/journal.pone.0308658)

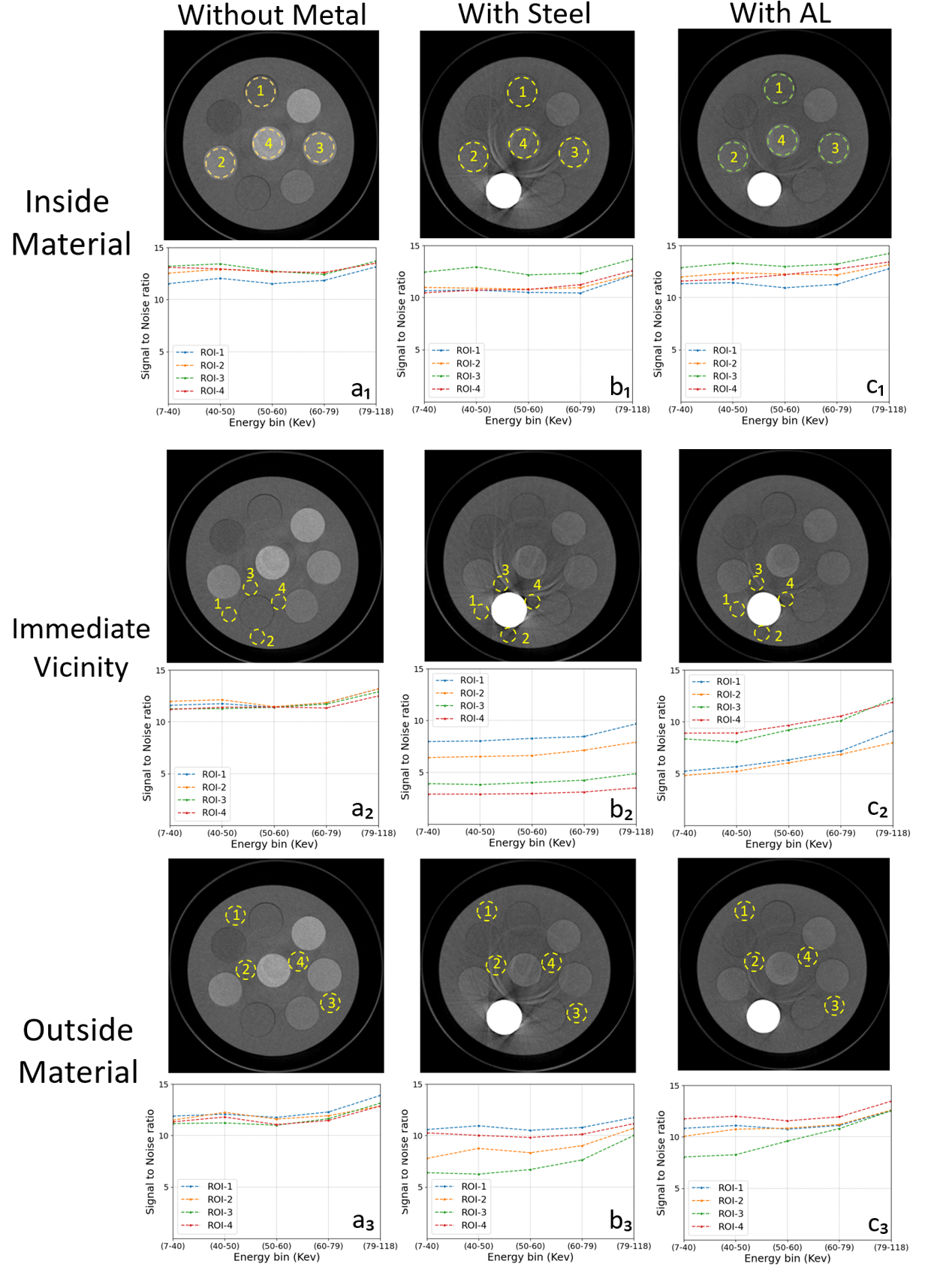

Supplement: S1 Fig — Signal-to-noise ratio (SNR) values for the case without any metal insert (a), with steel (b), and with aluminum (c) divided into three groups; inside the material(a1,b1,c1), immediate vicinity (a2, b2, c2), and outside material (a3, b3, c3). SNR values in the immediate vicinity for steel and aluminum were observed statistically significant (p<0.05). (TIF) [file pone.0308658.s001.tif]

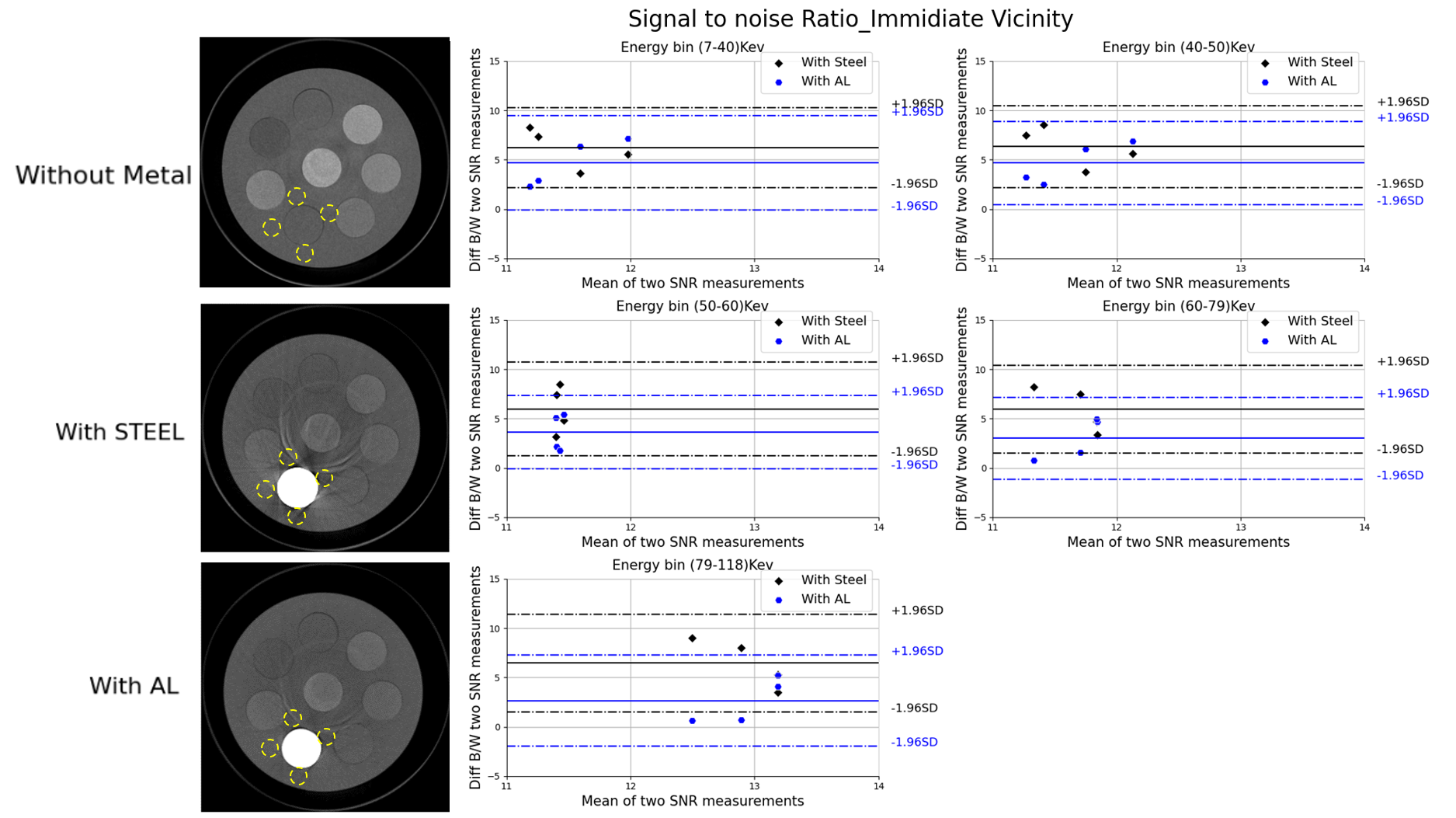

Supplement: S2 Fig — Bland-Altman plots showing the difference between SNR values as a function of the mean SNR values for the datasets of aluminum and steel inserts as compared to reference data (without any metal insert); for the case of the immediate vicinity of the metal object across five energy bins. The red and blue line represents the bias value, and the dashed lines represent the upper and lower limits of the mean values for steel and aluminum, respectively (confidence limits ± 1.96 × standard deviation). (TIF) [file pone.0308658.s002.tif]

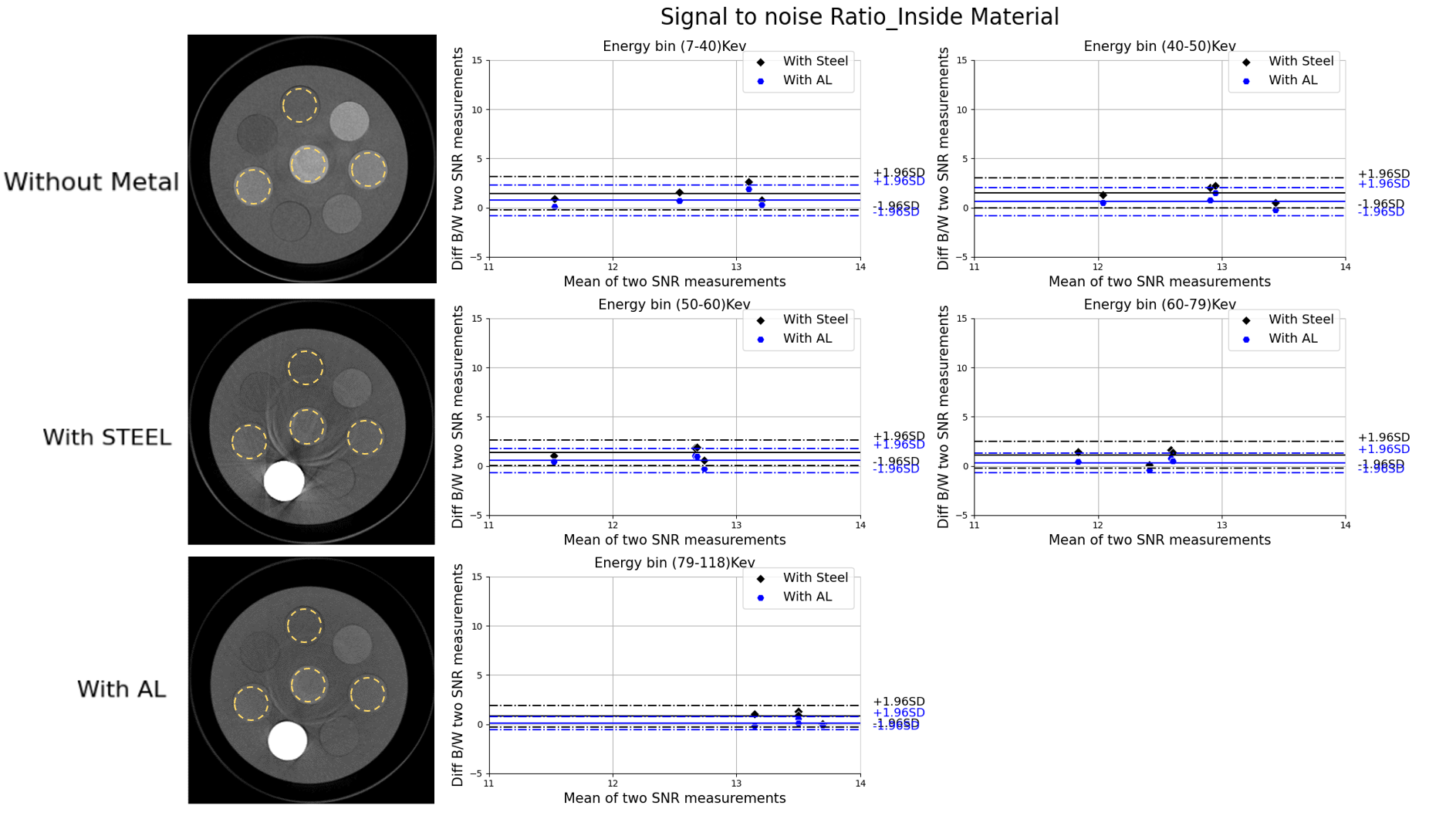

Supplement: S3 Fig — Bland-Altman plots showing the difference between SNR values as a function of the mean SNR values for the datasets of aluminum and steel inserts as compared to reference data (without any metal insert); for the case of inside the material across five energy bins. The red and blue line represents the bias value, and the dashed lines represent the upper and lower limits of the mean values for steel and aluminum, respectively (confidence limits ± 1.96 × standard deviation). (TIF) [file pone.0308658.s003.tif]

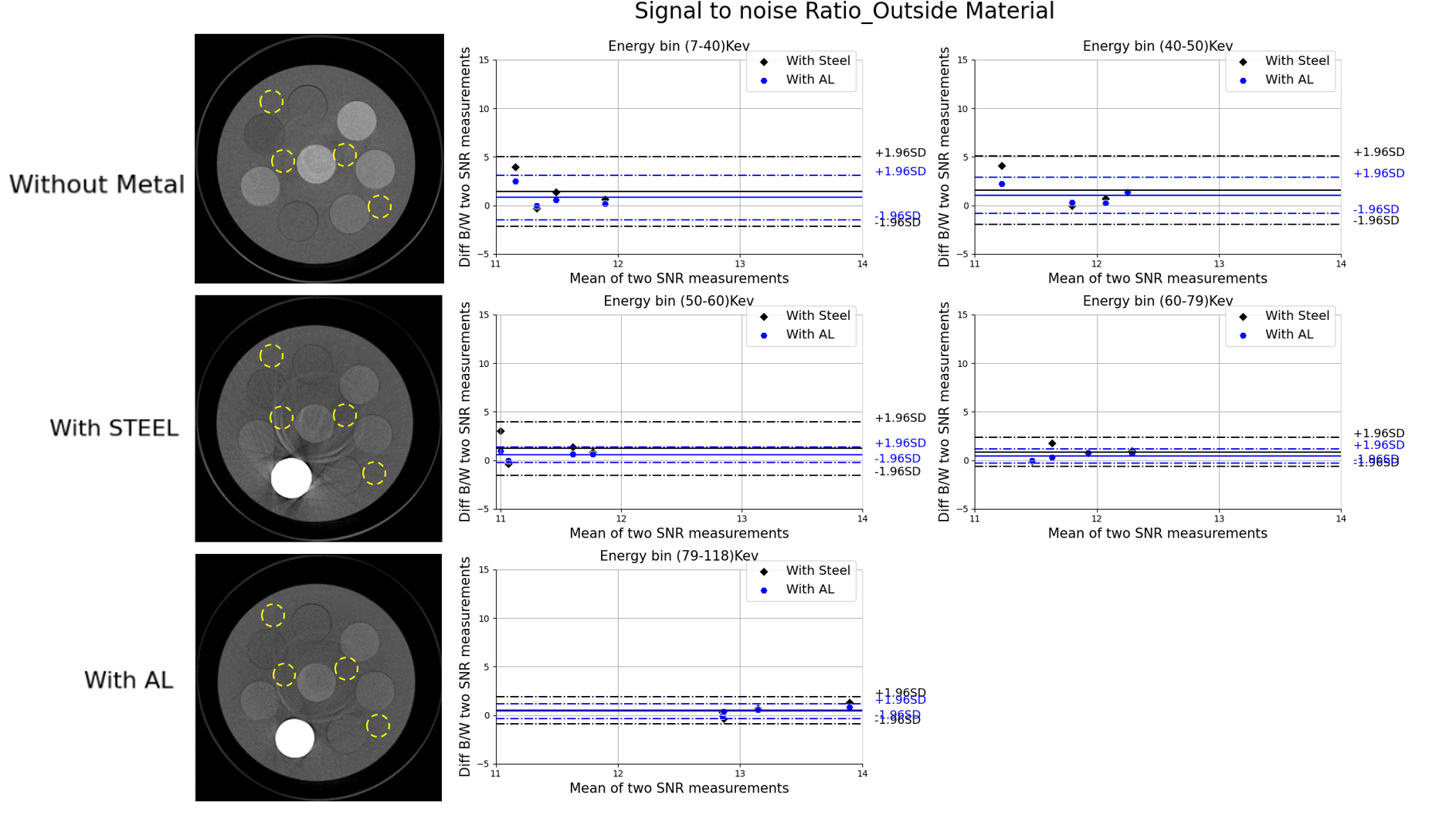

Supplement: S4 Fig — Bland-Altman plots showing the difference between SNR values as a function of the mean SNR values for the datasets of aluminum and steel inserts as compared to reference data (without any metal insert); for the case of outside the material across five energy bins. The red and blue line represents the bias value, and the dashed lines represent the upper and lower limits of the mean values for steel and aluminum, respectively (confidence limits ± 1.96 × standard deviation). (TIF) [file pone.0308658.s004.tif]
